# Sphingolipids as a new factor in the pathomechanism of preeclampsia – Mass spectrometry analysis

**DOI:** 10.1371/journal.pone.0177601

**Published:** 2017-05-19

**Authors:** Karol Charkiewicz, Joanna Goscik, Agnieszka Blachnio-Zabielska, Grzegorz Raba, Agata Sakowicz, Jaroslaw Kalinka, Adrian Chabowski, Piotr Laudanski

**Affiliations:** 1 Department of Perinatology and Obstetrics, Medical University of Bialystok, Bialystok, Poland; 2 Faculty of Computer Science, Bialystok University of Technology, Bialystok, Poland; 3 Department of Hygiene, Epidemiology and Metabolic Disorders, Bialystok, Poland; 4 Department of Physiology, Medical University of Bialystok, Bialystok, Poland; 5 Institute of Obstetric and Emergency Medicine, University of Rzeszow, Żurawica, Podkarpackie, Poland; 6 Department of Medical Biotechnology, Medical University of Lodz, Lodz, Poland; 7 Department of Perinatology, Medical University of Lodz, Lodz, Poland; Weizmann Institute of Science, ISRAEL

## Abstract

**Objective(s) and design:**

The aim of the study was to analyse a panel of 11 sphingolipids in plasma and three blood fractions (platelet-poor plasma, platelets and red blood cells) of women with mild preeclampsia.

**Materials and methods:**

We recruited 21 women between 25–40 weeks gestation with diagnosed mild preeclampsia to the study group and 36 healthy women with uncomplicated pregnancies, who corresponded with the study group according to gestational age, to the control group. To assess the concentration of 11 sphingolipids in the blood plasma and blood fractions, we used ultra-high performance liquid chromatography coupled with triple quadrupole mass spectrometry (UHPLC/MS/MS).

**Results:**

We showed a significant increase in the concentration of eight sphingolipids in the plasma of women with preeclampsia in comparison to the control group: Sph (p = 0.0032), S1P (p = 0.0289), C20-Cer (p < 0.0001), C18-Cer (p < 0.0001), C16-Cer (p = 0.012), C18:1-Cer (p = 0.003), C22-Cer (p = 0.0071), and C24:1-Cer (p = 0.0085).

**Conclusion:**

We showed that selected sphingolipids, especially C20-Cer and C18-Cer, are totally new factors in the pathomechanism of PE and that these bioactive lipids may play an important role in apoptosis and autophagy.

## Introduction

Preeclampsia (PE) is a disorder occurring in 3–5% of pregnancies in Western Europe and North America, with almost 8.5 million cases per year recorded worldwide [1]. It is the most common cause of mortality in pregnant women. Clinically, this disease is associated with hypertension ≥140/90 mm Hg and proteinuria ≥0.3 g/24 h, occurring after the 20th gestational week in women previously identified as normotensive and with no protein in their urine [2]. The course of preeclampsia is individually specific; it may present with varying degrees of severity of hypertension and proteinuria and may be complicated by the HELLP syndrome (haemolytic anaemia, elevated liver enzymes, low platelet count) and fully fledged eclampsia [3]. The symptoms are associated with generalized oedema, headache and blurred vision, and in severe cases, preeclampsia may cause liver failure and kidney disease, coagulation disorders, respiratory distress syndrome and intrauterine foetal growth restriction (IUGR) [2,4]. Despite many hypotheses, the pathogenesis of preeclampsia has not been clearly established, and the most effective ‘remedy’ is delivery [5].

In the literature, the most popular studies discuss the coexistence of metabolic syndrome and PE [6]. Different authors report an indirect relationship between PE and the increase of free fatty acids in patient’s blood. If we take into account the fact that increased levels of saturated fatty acids stimulate intracellular synthesis of sphingolipids, it can be postulated that sphingolipids are the element that link lipid abnormalities and preeclampsia [7]. Additionally, it is well known that IL-6 and TNF-alpha as well as MCP-1 and PAI-1 stimulate lipolysis and thus the release of free fatty acids from fat; free fatty acids are substrates for the synthesis of sphingolipids [8,9]. Additional relationships may exist between fatty acids and, e.g., triacylglycerols, diacylglycerols and neutral lipids, which are substrates for the synthesis of biological active lipids [10]. Specifically, triglycerides (TG) are the one of the most important type of lipids in PE. It was recently shown that amongst women with mild PE, the concentration of TG significantly increased in comparison to healthy pregnant women, which led to the understanding of the relationship between fatty acids and sphingolipid metabolism and their influence on lipotoxicity in PE [11,12].

The most recent research on the metabolic syndrome (MetS) revealed that MetS represents an oligogenic cluster of genetic factors and environmental metabolic overload / sedentary lifestyle, which includes hypertension, hyperlipidaemia, hyperglycaemia, insulin resistance, obesity, hyperuricaemia and other related clinical phenotypes [13]. Scientists explained the significance of the cluster of genetic factors, the regulation of food intake and energy consumption by genetic and environmental factors and their relation to the aetiology of severe obesity and MetS [14,15]. Additionally, scientists used GWAS (genome-wide association study) research for relating genetic factors (participants had their whole genome sequenced) to the influence of factors such as environment and lifestyle to determine the overall view for the pathogenesis of the disease [16].

However, in contrast, in our research, we intend to show that sphingolipids are also involved in the pathological mechanism of PE in patients who are not obese and do not have metabolic syndrome. It is worth noting that sphingolipids are not the primary cause of PE. In the literature, it is suggested that the above-mentioned cytokines and lipids can be involved in the molecular mechanism initiated by the maternal immunology response to the foetal portion of the placenta. Immune system activation is associated with the origin of PE and other factors, including chemokines, activated neutrophils, and endothelial dysfunction. We believe that through this mechanism, a disturbance in biologically active lipid levels is also related to the pathophysiology of this syndrome [10,17].

Romanowicz et al. discovered that in the umbilical artery, during preeclampsia, the levels of sphingosine and sphingomyelin increase and the level of ceramide content as well as sphingoid bases decrease, which may confirm the hypothetical change in the level of sphingolipids in the blood. Perhaps sphingolipids, whose content increases, are released into the mother’s blood through the placenta [18]. In addition, it was discovered that one sphingolipid, i.e., sphingosine-1-phosphate, inhibits the differentiation of cytotrophoblasts in the syncytiotrophoblast, which may be important in the pathogenesis of PE [19].

The relationship between higher levels of blood sphingolipids and hypertension is discussed in the literature [20,21]. These studies reveal that some sphingolipids, e.g., sphingosine-1 phosphate (S1P), play an important role in proliferation, cell growth, cell survival, migration, inflammation, angiogenesis, vasculogenesis and resistance to apoptotic cell death.

Because of the poorly researched topic of sphingolipids in PE and existence of strong evidence on the importance of these compounds in the pathogenesis of PE, we aimed to quantitatively examine their contents in the peripheral blood of pregnant women with a particular emphasis on sphingosine-1 phosphate.

## Materials and methods

In our Department (Perinatology and Obstetrics of Medical University of Bialystok) and two other Departments (Institute of Obstetric and Emergency Medicine of University of Rzeszow and Department of Perinatology of Medical University of Lodz), we eventually recruited 21 pregnant women with mild preeclampsia (study group) and 36 pregnant women (matched for maternal age, gestational age and BMI) with uncomplicated pregnancies (control group). The study protocol was approved by the Local Ethical Committee of Medical University of Bialystok, Poland, and informed consent was obtained from each patient (no ethics committee approval: R-I-002/377/2016). Signed informed consent was obtained from all participants involved in the study.

The recruitment of patients to the study and control groups started after 24 weeks of gestation because we attempted to perform OGTT 75 g (routinely conducted in Poland between the 24th and 28th weeks) in all of the patients. We recruited patients with mild preeclampsia between 25–40 weeks of gestation (patients with severe PE were not included because we focused on a higher homogeneity of the study group). The inclusion criteria were as follows: blood pressure between 140/90 and 160/110 mmHg in two independent measurements taken over an interval of at least 6 hours and the presence of protein in 24 hour urine collection above 300 mg/24 h, but not more than 5 g/24 h. We excluded women with: chronic hypertension, multiple pregnancy, pre-existing diabetes or gestational diabetes, insulin resistance, abnormal glucose and insulin fasting level, connective tissue disease, kidney disease, viral diseases (CMV, WR, EBV), toxoplasmosis, urinary tract infection, thrombocytopenia and coagulation disorders, pregnancy diagnosed with chromosomal aberrations before or after childbirth, and BMI > 30 at the time of recruitment. We obtained 20 ml of blood in EDTA tubes from each patient (fasting) qualified for the project.

In accordance with the model protocol of blood fractionation [22] repeatedly tested on rats, we obtained plasma and three blood fractions (platelet-poor plasma, platelets and red blood cells) for the determination of sphingolipids. This protocol was selected because we had previously tested it for compatibility with our sphingolipid extraction method. EDTA was used as an anticoagulant. Immediately after sampling, 20 ml of blood was centrifuged at 1400 × *g* for 10 min at 4°C and platelet-rich plasma was transferred to a fresh plastic tube. The leukocyte-rich buffy coat was thoroughly removed. Separated erythrocytes were suspended in 3 ml of cold PBS buffer (pH 7.4) and centrifuged at 1400 × *g* for 10 min. The upper layer and remaining buffy coat were discarded. Erythrocytes were then re-suspended in 2 ml of PBS buffer (pH 7.4) and were flash frozen in liquid nitrogen. Platelet-rich plasma was centrifuged at 2000 × *g* for 10 min at 4°C to isolate platelets. Isolated platelets were washed with cold platelet wash buffer (5 mM KH_2_PO_4_, 5 mM Na_2_HPO_4_, 0.1 M NaCl, 1% glucose, 0.63% sodium citrate, pH 6.6), suspended in 0.3 ml of PBS, and flash frozen in liquid nitrogen. The supernatant was then transferred to a fresh plastic tube and centrifuged at 5000 × *g* for 10 min to obtain platelet-free plasma. All samples were stored at −80°C until analysis [22]. Platelet and erythrocyte fractions, before sphingolipid extraction, were gently sonicated. Then, each sample was divided into two portions: one for the extraction sphingolipids, the second for the determination of total protein. Therefore, the ceramide content in the fraction of platelets, red blood cells were standardized. The concentrations of the sphingolipid fractions were converted to nmol/mg of total protein (platelets) and ng/mg of haemoglobin (erythrocytes). The protein was measured by absorbance using reagents; the Thermo Scientific Pierce BCA Protein Assay Kit was used to measure total protein and Drabkin's reagent was used to measure haemoglobin.

The content of sphingolipids was measured using a UPLC/MS/MS in multiple reaction monitoring (MRM) mode according to Blachnio-Zabielska et al. [23–25]. The method uses an internal standard approach with individual concentration curves prepared with the use of commercially available sphingolipid standards (Avanti Polar Lipids). Briefly, 50 μl of the internal standard solution (17C-sphingosine and 17C-S1P, and C17-Cer Avanti polar lipids) was added to each sample (100 μl), as well as 1.5 ml of an extraction mixture (isopropanol:water:ethyl acetate, 35:5:60; v:v:v). The following sphingolipids were quantified: Sph (sphingosine), S1P (sphingosine-1-phosphate), SPA (sphinganine), ceramide C14:0-Cer (ceramides containing myristic acid), C16:0-Cer (ceramides containing palmitic acid), C18:1-Cer (ceramides containing oleic acid), C18:0-Cer (ceramides containing stearic acid), C20:0-Cer (ceramides containing arachidic acid), C22:0-Cer (ceramide containing behenic acid), C24:1-Cer (ceramides containing nervonic acid) and C24:0-Cer (ceramides containing lignoceric acid). Sphingolipids were analysed by means of an Agilent 6460 triple quadrupole mass spectrometer using a positive ion electrospray ionization (ESI) source with multiple reaction monitoring (MRM). Chromatographic separation was performed using an Agilent 1290 Infinity Ultra Performance Liquid Chromatography (UPLC). The analytical column was a reverse-phase Zorbax SB-C8 column 2.1 × 150 mm, 1.8 μm. Chromatographic separation was conducted in a binary gradient using 2 mM ammonium formate, 0.15% formic acid in methanol as Solvent A and 1.5 mM ammonium formate and 0.1% formic acid in water as Solvent B at a flow rate of 0.4 ml/min. HPLC grade methanol, water, formic acid, ammonium formate and ethanol were purchased from Sigma-Aldrich (St. Louis, MO).

Descriptive statistics, including the mean concentration, standard error of the mean and median, were calculated for the sphingolipids under investigation, henceforth called features. To determine whether the features’ distributions significantly differed between the studied groups, either Student’s t-test was carried out or a non-parametric Wilcoxon rank-sum test [26] was applied. The choice of an appropriate method was made upon fulfilling the normality and homogeneity of variances assumptions, and in the case of a violation of at least one of the conditions, a non-parametric approach was employed. The normality of the distribution of the features was checked with the Shapiro-Wilk test [27], and the homogeneity of variances was checked with Levene’s test [28].

## Results

The clinical characteristics of the patients are presented in Table 1. Patients from both groups were matched for maternal age, number of pregnancies, gestational age at collection and present BMI to ensure that the two groups are comparable and there are no statistically significant differences between them.

**Table 1:** Clinical patient characteristics.

	Group I—healthy, pregnant women (n = 36)	Group II—women with PE (n = 21)
Maternal age (median ± SD)	23.2 ± 6.01	29 ± 5.65
Number of pregnancies (median ± SD)	1 ± 1.27	1 ± 0.56
Gestational age at sample collection in weeks (median ± SD)	31.6 ± 5.78	32.5 ± 4.97
Present BMI (median ± SD)	25.09 ± 2.04	26 ± 2.53

SD—standard deviation

The values of the mean sphingolipid concentration and standard error (mean ± SEM) of maternal plasma as well as of three fractions of blood (platelet-poor plasma, platelet, red blood cells) in each study group are presented in Tables 2 to 5.

**Table 2:** Concentrations of sphingolipids in maternal plasma.

	Group I—healthy, pregnant women (n = 36)	Group II—women with PE (n = 21)	P-value
	Sphingolipids concentration (nmol/l) Mean ± SEM		Group I- Group II
SPA	36.18 ± 2.99	39.83 ± 3.32	0.33**
**Sph**	129.3 ± 10.02	209.15 ± 28.4	0.0033** ^
**S1P**	176.38 ± 13.46	269.97 ± 38.23	0.028** ^
C24-Cer	2192.05 ± 107.35	2309.56 ± 177.58	0.57*
**C20-Cer**	161.98 ± 6.57	225.85 ± 11.97	< 0.0001** ^
**C18-Cer**	134.44 ± 7.43	192.46 ± 9.55	< 0.0001** ^
**C16-Cer**	675.41 ± 21.96	793.41 ± 35.36	0.012* ^
C14-Cer	20.62 ± 0.98	21.01 ± 1.18	0.57**
**C18:1-Cer**	16.51 ± 0.71	21.13 ± 0.89	0.003* ^
**C22-Cer**	898.28 ± 43.29	1183.33 ± 78.55	0.0071* ^
**C24:1-Cer**	1885.99 ± 83.89	2338.48 ± 124.67	0.0085* ^

* p value calculated using Student’s T-test

** p value calculated using Mann Whitney Wilcoxon’s test

^ statistically significant p value of less than 0.05 (p < 0.05)

**Table 3:** Concentrations of sphingolipids in the blood fraction: Platelet-poor plasma.

	Group I—healthy, pregnant women (n = 36)	Group II—women with PE (n = 21)	P-value
	Sphingolipids concentration (nmol/l) Mean ± SEM		Group I- Group II
SPA	7.3 ± 1	6.31 ± 0.66	0.47*
Sph	17.71 ± 2	15.04 ± 1.34	0.38*
S1P	43.76 ± 5.01	64.86 ± 12.39	0.68**
C24-Cer	293.7 ± 21.56	258.59 ± 36.19	0.41*
C20-Cer	20.73 ± 1.85	21.07 ± 2.7	0.91**
C18-Cer	16.63 ± 1.59	17.69 ± 1.95	0.68*
C16-Cer	86.17 ± 7.07	91.2 ± 7.63	0.63*
C14-Cer	2.75 ± 0.2	2.55 ± 0.39	0.23**
C18:1-Cer	1.95 ± 0.18	2.13 ± 0.23	0.72*
C22-Cer	114.28 ± 9.34	119.11 ± 16.26	0.88**
C24:1-Cer	229.72 ± 16.99	249.34 ± 26.26	0.81**

* p value calculated using Student’s T-test

** p value calculated using Mann Whitney Wilcoxon’s test

**Table 4:** Concentrations of sphingolipids in the blood fraction: Platelets.

	Group I—healthy, pregnant women (n = 36)	Group II—women with PE (n = 21)	P-value
	Sphingolipids concentration (nmol/mg of total protein) Mean ± SEM		Group I- Group II
SPA	0.02 ± 0.002	0.02 ± 0.003	0.97*
Sph	0.07 ± 0.02	0.06 ± 0.01	0.56*
S1P	0.35 ± 0.08	0.36 ± 0.06	0.96*
C24-Cer	0.11 ± 0.01	0.09 ± 0.01	0.31*
C20-Cer	0.06 ± 0.012	0.06 ± 0.008	0.85**
C18-Cer	0.03 ± 0.0052	0.03 ± 0.0053	0.91*
C16-Cer	0.08 ± 0.013	0.08 ± 0.014	0.97**
C14-Cer	0.002 ± 0.0001	0.002 ± 0.0004	0.69**
C18:1-Cer	0.002 ± 0.0002	0.002 ± 0.0004	0.73*
C22-Cer	0.17 ± 0.03	0.13 ± 0.02	0.54**
C24:1-Cer	0.14 ± 0.02	0.11 ± 0.01	0.59**

* p value calculated using Student’s T-test

** p value calculated using Mann Whitney Wilcoxon’s test

**Table 5:** Concentrations of sphingolipids in the blood fraction: Red blood cells.

	Group I—healthy, pregnant women (n = 36)	Group II—women with PE (n = 21)	P-value
	Sphingolipids concentration (nmol/mg of Haemoglobin) Mean ± SEM		Group I- Group II
SPA	0.0004 ± 0.00003	0.0004 ± 0.00007	0.94*
Sph	0.0004 ± 0.00007	0.0004 ± 0.00003	0.72**
S1P	± 0.0016	0.008 ± 0.0009	0.12**
C24-Cer	0.008 ± 0.001	0.009 ± 0.001	0.33**
C20-Cer	0.0008 ± 0.0001	0.0009 ± 0.0001	0.86*
C18-Cer	0.001± 0.0001	0.002 ± 0.0002	0.42*
C16-Cer	0.007 ± 0.0005	0.006 ± 0.0004	0.42*
C14-Cer	0.00008 ± 0.000011	0.00008 ± 0.000013	0.79**
C18:1-Cer	0.0001 ± 0.00001	0.0001 ± 0.00002	0.39**
C22-Cer	0.004 ± 0.0005	0.006 ± 0.0007	0.15*
C24:1-Cer	0.03 ± 0.0029	0.03 ± 0.0024	0.96*

* p value calculated using Student’s T-test

** p value calculated using Mann Whitney Wilcoxon’s test

We showed a significant increase in the concentration of eight sphingolipids in the plasma of women with preeclampsia in comparison to the control group (Table 2): Sph (p = 0.0032), S1P (p = 0.0289), C20-Cer (p < 0.0001), C18-Cer (p < 0.0001), C16-Cer (p = 0.012), C18:1-Cer (p = 0.003), C22-Cer (p = 0.0071), and C24:1-Cer (p = 0.0085).

There are no significant differences between groups in the three blood fractions: platelet-poor plasma, platelet, red blood cells (Tables 3–5).

## Discussion

Sphingolipids, especially ceramides, are biologically active lipids that are involved in many metabolic processes in the human body. One of their functions is signal transduction in apoptosis and cell autophagy [29,30]. It is known that in the pathogenesis of PE, a decisive role is played by abnormal implantation of the trophoblast in the uterine spiral arteries, leading to placental ischemia and oxidative stress, which consequently results in cell apoptosis, the release of placental factors (including inflammatory proteins), and an imbalance between pro- and anti-angiogenic factors [31–33]. Because of oxidative stress and apoptosis in preeclampsia, it can be assumed that sphingolipids should play a significant role in the pathogenesis of PE.

Interestingly, our findings indicate no significant differences in sphingolipid levels among the three blood fractions. Before the experiment, we hypothesized that sphingolipids can be released from the placenta into the blood of women with PE [34–36]. This indicates a lack of increased synthesis of sphingolipids in blood platelets and erythrocytes, tentatively confirming our hypothesis about the placental source of sphingolipids.

This theory seems to be confirmed by another study by Melland-Smith et al. [37], who found an increased amount of ceramides in the placenta of women with preeclampsia [37]. Their analysis showed significant increases in various ceramides, except for C22-Cer, in PE placentas compared to control group placentas: C16-Cer, C18-Cer, C20-Cer and C24-Cer [37]. The same result, in a study of the ceramide content in the placenta of women with PE, was obtained by others [38]. Interestingly, Romanowicz et al. [18] showed a statistically significant decrease of ceramides levels in the umbilical cord artery; in particular, they showed a decrease of the total amount of ceramides and a decrease in the levels of individual ceramides, such as C12-Cer, C14-Cer, C16-Cer, C18-Cer, C14:1-Cer, C18:2-Cer, C20:4-Cer, and C20:5-Cer. In addition, they found an increase in the level of sphinganine in umbilical arteries in women with preeclampsia [18]. One year later, Romanowicz et al. [39] found an interesting result in research on the content of sphingolipids in Wharton's jelly, the substance surrounding umbilical cord vessels to protect them against extension, bending, twisting and compression. They showed an increase in the level of ceramide, sphingosine, sphinganine and sphingosine 1-phosphate in Wharton's jelly of umbilical cords in women with PE compared to the control group [39]. These studies are actually not in opposition to those mentioned above because it can be assumed that there is some pathomechanism that links the increase of sphingolipid content in placental tissue, decrease in umbilical artery and increase in Wharton's jelly. This relationship should be examined in detail in the future. It is worth noting that the relationship of sphingolipids in different parts of the placenta is not direct because the umbilical cord artery and Wharton's jelly are not directly connected with spiral arteries.

In the international scientific literature, there is not much research on sphingolipids in the plasma of women with PE; this is a very poorly studied topic. In our research, we demonstrated a statistically significant increase in eight sphingolipids in the plasma of women with PE: Sph, S1P, C20-Cer, C18-Cer, C16-Cer, C18:1-Cer, C22-Cer, and C24:1-Cer. Our results confirmed the data obtained in other research, revealing elevated ceramides levels, including C16-Cer, C18-Cer, C20-Cer and C24-Cer, in the plasma of PE patients relative to those found in control normotensive women [37]. However, these studies showed a decrease in S1P in the serum of women with PE, which is contrary to that found in our study showing an elevated level of S1P in the plasma of women with PE.

Sigruener et al. showed results from a large cohort—LURIC study (3600 individuals)–on the mortality and morbidity in relation to individual sphingolipid species. It was shown that the differences in the lipotoxicity of individual short-chain ceramide species and their resemblance to short chain saturated fatty acids are responsible for endoplasmic reticulum stress, mitochondrial damage and subsequent activation of autophagy and apoptosis. Scientists revealed that five Cer species were significantly associated with mortality: three showed a positive association with mortality (16∶0, 18∶0, 24∶1) and the remaining two Cer species (23∶0, 24∶0) were slightly protective. Phosphatidylcholine (PC) 32∶0 (probably PC 16∶0/16∶0) and sphingomyelin (SM 16∶0) together with Cer 24∶1 showed the strongest positive association with mortality. Interestingly, similar tendencies were observed for 16∶0, 23∶0, 24∶0 and 24∶1 SM and Cer species. These results clearly show that ceramides play a major role in lipotoxicity [40].

As mentioned above, sphingolipids play an important role in apoptosis, oxidative stress and cell autophagy; therefore, our results showing increased levels of long-chain ceramides confirm this theory. Young et al. noted that Sph, C16, C18, and C20 ceramides play a vital role in antiproliferative processes and C16 and C18 ceramides are also proapoptotic. Interestingly, some sphingolipids, such as S1P, act contrary to long-chain ceramides and "switch” autophagy towards cell survival through the activation of ERK and suppression of ceramide-induced JNK activation [30,41]. Thus, the elevated levels of S1P in PE could be a compensating and counteracting mechanism for proapoptotic ceramides. Moreover, ceramide C24: 1 has a proliferative effect; therefore, it may act similar to S1P (a compensatory effect) [42]. S1P also induces the activation of inflammatory mediators, such as VCAM-1, ICAM-1 and COX, which are elevated in both blood and placenta during preeclampsia [43–45]. Furthermore, a study conducted by Seki et al. [46] on female C57BL/6 mice implicates (the role of) S1P in the process of Th1 and Th17 cell migration. Both cell fractions are elevated during pregnancies complicated by hypertension and proteinuria [47]. Sph, C16, C18, and C20 ceramides activate apoptosis through a mitochondrial pathway involving the proapoptotic Bax protein, however sphingosine activates the lysosome apoptosis pathway first [30]. Furthermore, scientists revealed a significant increase in the level of C24:1 and C24:0 ceramides in plasma in humans with hypertension, additionally suggesting their role in PE [20].

Sphingolipids have also been implicated in the endothelium-dependent release of tromboxane A2 (TXA2), contributing to endothelial dysfunction and elevation of arterial blood pressure [20]. The elevated level of TXA2 is one of the biochemical markers of preeclampsia development [48]. Chen et al. found an increased concentration of TXA2 (role of activation of platelet aggregation) in the plasma of women with PE and a decrease in the concentration of prostacyclin PGI2 (role of vasodilation and inhibition of platelet aggregation) [49]. S1P is primarily produced by blood platelets, and platelet aggregation stimulated by TXA2 may result in thrombocytopenia [49]. In contrast to our study, Melland-Smith et al. showed reduced levels of S1P in the serum of women with PE; this finding can be explained by the low platelet count in women with PE [37]. However, such a mechanism does not always exist because researchers found that in mild PE (studied by our team), thrombocytopenia does not always occur [50].

Moreover, ceramides, which are among the most important sphingolipids, are involved in growth inhibition and apoptosis of cardiac and vascular tissues because of their influence on angiotensin II type 2 receptors, which have a role in blood pressure regulation [51,52].

In this publication, we showed that selected sphingolipids may play a role in the pathomechanism of apoptosis and autophagy in PE. In the international literature, not enough relevant research has focused on the role of sphingolipids in the pathogenesis of this disease. However, because of the complexity of the pathomechanism responsible for preeclampsia, further functional experiments should be performed.

## Supporting information

S1 File Raw data of sfingolipid concentration in plasma and 3 fractions, presented in ng/100ul. (XLSX)

S2 File ANOVA analysis of results in plasma. (XLSX)

S3 File ANOVA analysis of results in erythrocytes. (XLSX)

S4 File ANOVA analysis of results in plasma fraction. (XLSX)

S5 File ANOVA analysis of results in platelets fraction. (XLSX)
